# Involvement of mucosal flora and enterochromaffin cells of the caecum and descending colon in diarrhoea-predominant irritable bowel syndrome

**DOI:** 10.1186/s12866-021-02380-2

**Published:** 2021-11-13

**Authors:** Jingze Yang, Peng Wang, Tong Liu, Lin Lin, Lixiang Li, Guanjun Kou, Ruchen Zhou, Pan Li, Yanqing Li

**Affiliations:** 1 Department of Gastroenterology, Qilu Hospital, Cheeloo College of Medicine, Shandong University, No. 107, Wenhuaxi Road, Jinan, 250012 Shandong China; 2 Laboratory of Translational Gastroenterology, Qilu Hospital, Cheeloo College of Medicine, Shandong University, No. 107, Wenhuaxi Road, Jinan, 250012 Shandong China; 3 Robot Engineering Laboratory for Precise Diagnosis and Therapy of GI Tumor, Qilu Hospital, Cheeloo College of Medicine, Shandong University, No. 107, Wenhuaxi Road, Jinan, 250012 Shandong China

**Keywords:** Gut microbiota, Enterochromaffin cell, Diarrhoea-predominant irritable bowel syndrome, caecum and descending colon

## Abstract

**Background:**

Accumulating evidence supports the pivotal role of intestinal flora in irritable bowel syndrome (IBS). Serotonin synthesis by enterochromaffin (EC) cells is influenced by the gut microbiota and has been reported to have an interaction with IBS. The comparison between the microbiota of the caecal and colonic mucosa in IBS has rarely been studied. The aim of this study was to investigate the relationship between the gut microbiota, EC cells in caecum and descending colon, and diarrhoea-predominant IBS (IBS-D) symptoms.

**Results:**

A total of 22 IBS-D patients and 22 healthy controls (HCs) were enrolled in our study. Hamilton anxiety (HAM-A) and Hamilton depression (HAM-D) grades increased significantly in IBS-D patients. In addition, the frequency of defecation in IBS-D patients was higher than that in HCs. Among the preponderant bacterial genera, the relative abundance of the *Ruminococcus_torques_ group* increased in IBS-D patients in caecum samples while *Raoultella* and *Fusobacterium* were less abundant. In the descending colon, the abundance of the *Ruminococcus_torques_group* and *Dorea* increased in IBS-D patients and *Fusobacterium* decreased. No difference was observed between the descending colon and caecum in regards to the mucosal-associated microbiota. The number of EC cells in the caecum of IBS-D patients was higher than in HCs and the expression of TPH1 was higher in IBS-D patients both in the caecum and in the descending colon both at the mRNA and protein level. Correlation analysis showed that the *Ruminococcus_torques_group* was positively associated with HAM-A, HAM-D, EC cell number, IBS-SSS, degree of abdominal pain, frequency of abdominal pain and frequency of defecation. The abundance of *Dorea* was positively associated with EC cell number, IBS-SSS, HAM-A, HAM-D and frequency of abdominal pain.

**Conclusions:**

EC cell numbers increased in IBS-D patients and the expression of TPH1 was higher than in HCs. The *Ruminococcus torques group* and *Dorea* furthermore seem like promising targets for future research into the treatment of IBS-D patients.

**Supplementary Information:**

The online version contains supplementary material available at 10.1186/s12866-021-02380-2.

## Background

Irritable bowel syndrome (IBS) is a functional gastrointestinal disorder, which affects approximately 3–5% of the adult population and severely affects the quality of life [1]. It is characterised by diverse symptoms, such as abdominal pain or distension, constipation, or diarrhoea. According to the bowel habit, IBS is divided into diarrhoea-predominant (IBS-D), constipation-predominant, mixed, with both diarrhoea and constipation, and unsubtyped IBS. However, current treatment approaches for IBS are only modestly effective because of the undefined pathogenesis, which is considered to be multifactorial [2].

IBS has been reported to be associated with dysbiosis [3–7], and probiotic therapies were beneficial to patients with IBS in previous studies [8–10]. For gut microbiota assessment, most studies selected faeces [5, 11, 12] as samples because of their convenience and accessibility. Nevertheless, mucosal samples [13–15] may be a better choice for microbiota research, which reflects the status of the microbial community of the organism. A systematic review summarized previous studies and suggested a lack of consistency among studies, particularly mucosal microflora assessment [16].

The intestinal microbiota plays a role in regulating serotonin (5-hydroxytryptamine, 5-HT) levels [17]. As an essential neurotransmitter, 5-HT is associated with altered motility and visceral discomfort and is involved in the pathogenesis of IBS [18]. Evidence from animal and clinical studies supports the key role of gut microbiota in mood regulation. On the one hand, germ-free (GF) mice showed fewer anxiety/depressive behaviours. However, when transplanted with the flora of patients with major depression, the depression phenotype was also inherited [19]. On the other hand, anxiety and depression behaviours can be alleviated by using probiotics. Bifidobacterium plays a role in anti-depression in a 5-HT dependent manner [20]. The vast majority of 5-HT is synthesized by enterochromaffin (EC) cells, one of the most abundant EC cells in the entire gastrointestinal tract, and stored in large dense core vesicles with acidic proteins such as chromogranin A (CgA). EC cells express various receptors which detect mechanical or chemical stimulations including pro-inflammatory mediators, bacterial metabolites, chemical irritants, and nutrients. A study revealed that intestinal EC cells were larger in GF rats than specific-pathogen-free rats, suggesting that the microbiota may influence the growth or function of EC cells [21].

**Fig. 1:**
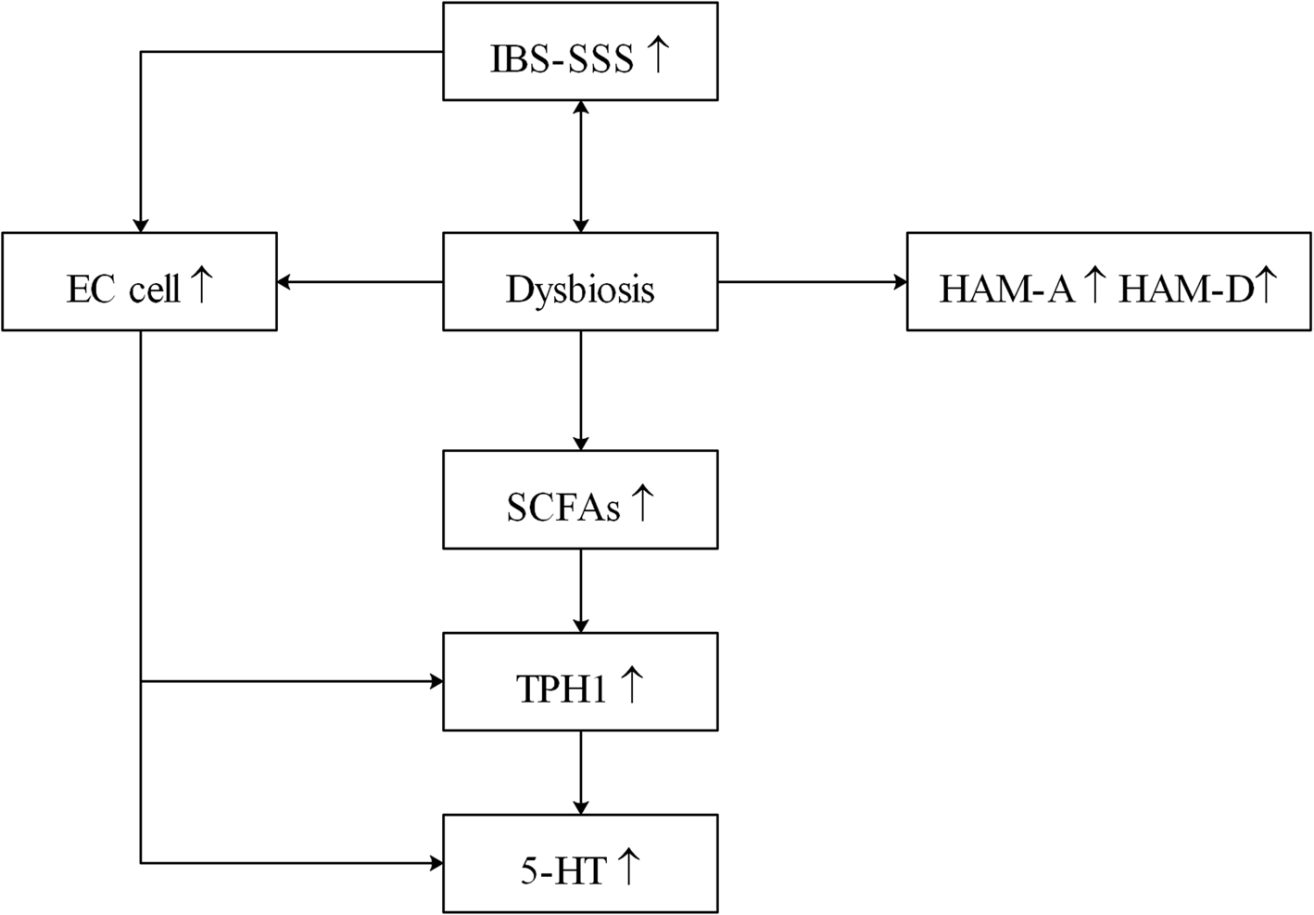
The overview figure

Intestinal flora have heterogeneous microbial communities in different segments of the gut in animals and the caecum, an ideal habitat for a variety of microorganisms, and is the most abundant part of the intestinal flora [22]. In clinical studies, by comparing the contents of the human caecum with faeces, Marteau et al. found a significant difference in the composition of bacteria [23]. In the caecum, the microbiota produces higher concentrations of short-chain fatty acids (SCFAs) and metabolites, which promote the production of 5-HT by increasing the expression of the rate-limiting enzyme tryptophan hydroxylase 1 (TPH1) [17]. However, the microbiota in the caecal mucosa of people has rarely been studied. With the stability of flora among individuals, the colonic mucosa is a good choice for microbiota research [24]. Accordingly, we aimed to investigate the difference in mucosal-associated microbiota and EC cells between the caecum and descending colon in IBS-D patients (Fig. 1).

## Results

### Clinical features of IBS-D patients and HCs

A total of 22 HCs and 22 IBS-D patients were recruited in this study, and the detailed information of the participants is presented in Table 1. There were no differences in sex, age and body mass index (BMI; *P* > 0.05) between the two groups. However, the Hamilton anxiety scale (HAM-A) scores, Hamilton depression scale (HAM-D) scores, and frequency of defecation were higher in IBS-D patients than in HCs (*P* <  0.0001).

**Table 1:** Clinical and demographic features of all study subjects

	IBS-D patients	HCs	*P*
Age, mean ± SD, years	(46.7 ± 10.8)	(44.4 ± 9.9)	0.469
Sex, male/female, n (%)	9/13(41/59)	15/7(68/32)	0.069
BMI	22.6 ± 2.3	24.1 ± 2.5	0.066
HAM-A	6.64 ± 3.71	1.36 ± 1.14	< 0.0001
HAM-D	4.00 ± 3.15	1.27 ± 0.98	< 0.0001
Frequency of defecation	4.59 ± 2.06	1.18 ± 0.39	< 0.0001
Degree of abdominal pain	50.00 ± 14.80	–	–
Frequency of abdominal pain	59.09 ± 21.80	–	–
Duration of disease	4.64 ± 2.72	–	–
IBS-SSS	194.55 ± 51.71	–	–

*BMI* Body mass index, *HAM-A* Hamilton anxiety scale, *HAM-D* Hamilton depression scale

### Microbiota diversity in IBS-D patients and HCs

Microbiota diversity was analyzed using conventional classical ecological descriptive approaches including alpha-diversity (richness as measured by the number of operational taxonomic units [OTUs] and assessed by Chao index) and beta-diversity metrics (principal coordinate analysis and Bray-Curtis distance using bacterial genus relative abundance). The results showed no difference in microbiota richness and microbiota variability between IBS-D patients and HCs (Additional file 1).

### Differences of mucosal-associated microbiota between IBS-D patients and HCs

**Fig. 2:**
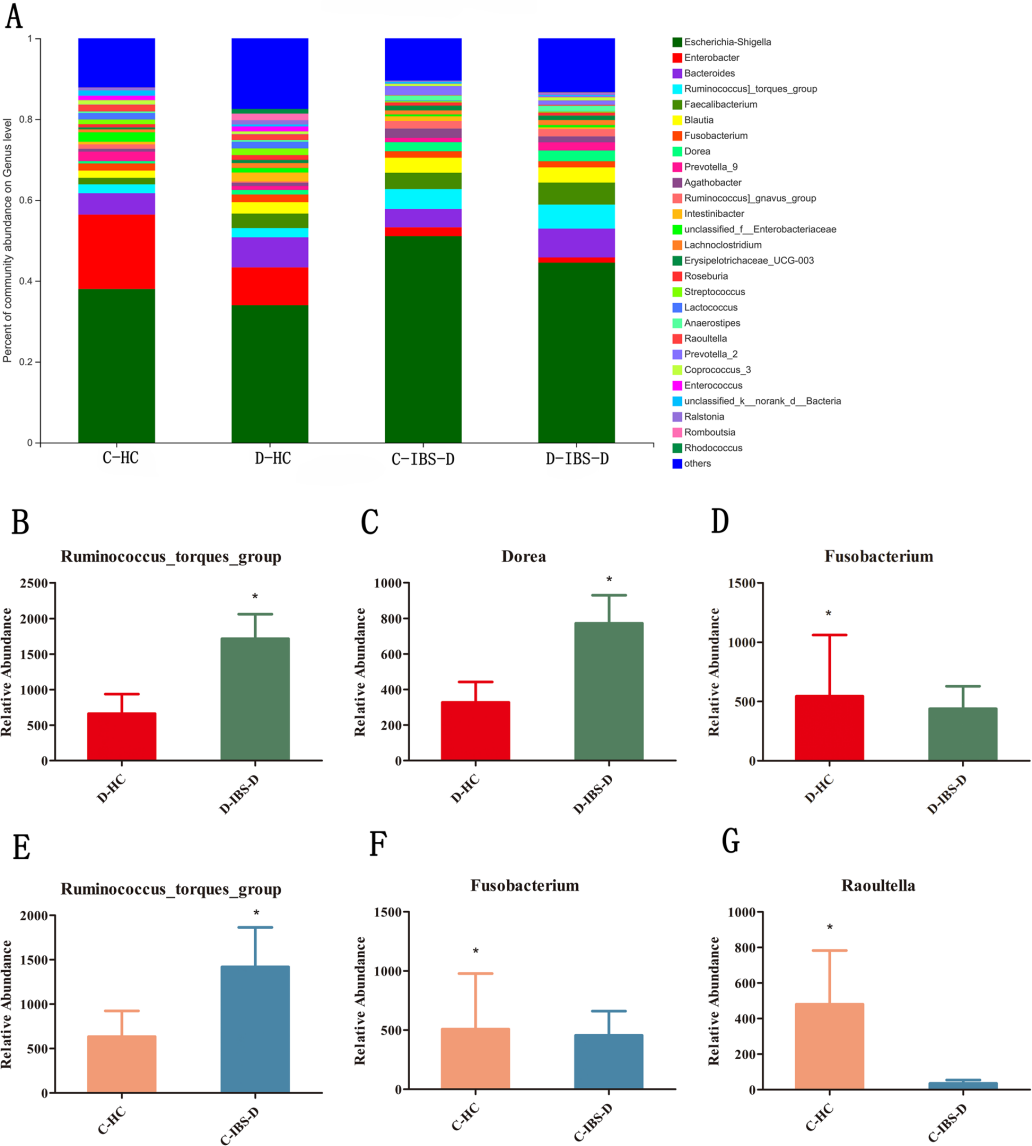
Relative abundances of genera in the descending colon and caecum of HCs and IBS-D patients. **a** Relative abundances of genera in the descending colon and caecum of HCs and IBS-D patients. **b**-**d** The comparison of *Ruminococcus_torques_group*, *Dorea*, and *Fusobacterium* between D-HCs and D-IBS-D patients. **e**-**g** The comparison of *Ruminococcus_torques_group*, *Raoultella* and *Fusobacterium* between C-HCs and C-IBS-D patients. (D-HC refers to the descending colon in HCs; D-IBS-D refers to the descending colon in IBS-D patients; C-HC refers to the caecum in HCs; C-IBS-D refers to the caecum in IBS-D patients) * *P* < 0.05

Community composition analysis showed that the common gut microbiota was Proteobacteria, Firmicutes, Bacteroidetes, Actinobacteria and Fusobacteria. However, there was no statistical difference between IBS-D patients and HCs at the phylum level (Additional file 2). Among the preponderant bacteria genera, the relative abundances of *Ruminococcus_torques_group* and *Dorea* were higher in the descending colon of IBS-D patients than in HCs (5.94% vs. 2.29%, *P* = 0.04183; 2.68% vs. 1.14%, *P* = 0.04962), whereas *Fusobacterium* decreased in patients with IBS-D (1.52% vs. 1.89%, *P* = 0.0345). Compared with the microbiota of the caecum in HCs, IBS-D patients showed an increased relative abundance of *Ruminococcus_torques_group* (4.91% vs. 2.20%, *P* = 0.04763) and decreased relative abundances of *Fusobacterium* and *Raoultella* (1.58% vs. 1.76%, *P* = 0.03117; 0.12% vs. 1.66%, *P* = 0.01892) (Fig. 2).

There were no differences in the levels of phyla and genera in the mucosal-associated microbiota between the descending colon and caecum (Additional files 2 and 3).

### EC cells number and TPH1 expression

**Fig. 3:**
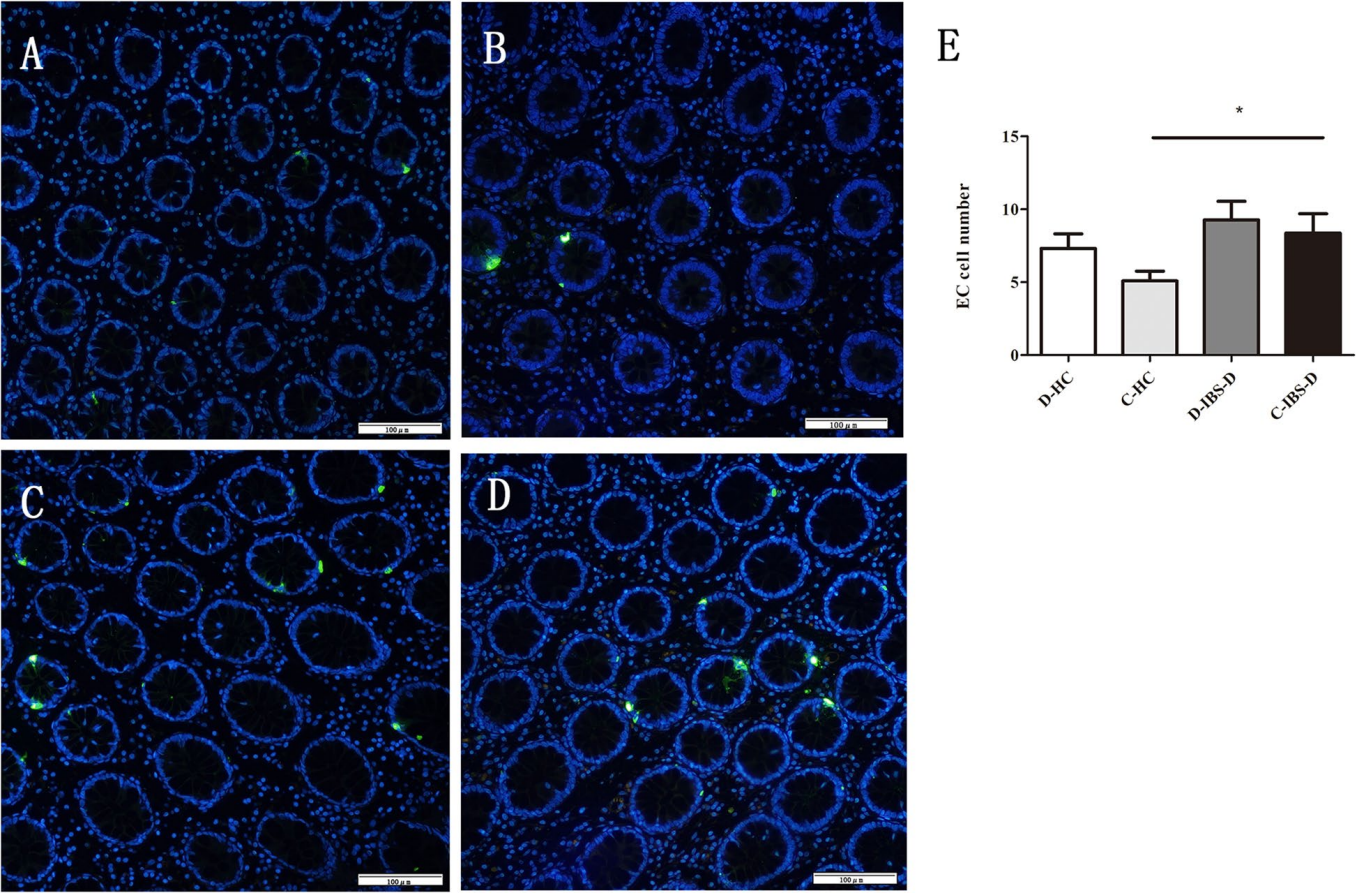
Immunofluorescence analysis of EC cells in the IBS-D patients and HCs. **a** Representative image of EC cells (green) in the descending colon of the HCs, 200×. **b** Representative image of EC cells (green) in the caecum of the HCs, 200×. **c** Representative image of EC cells (green) in the descending colon of the IBS-D patients, 200×. **d** Representative image of EC cells (green) in the caecum of the IBS-D patients, 200×. **e** The number of EC cells in the descending colon and caecum in the HCs and IBS-D patients and the number of EC cells of the caecum in IBS-D patients was more than that in HCs. The data are presented as mean ± SD. (D-HC refers to the descending colon in HCs, D-IBS-D refers to the descending colon in IBS-D patients, C-HC refers to the caecum in HCs, C-IBS-D refers to the caecum in IBS-D patients). * *P* < 0.05

**Fig. 4:**
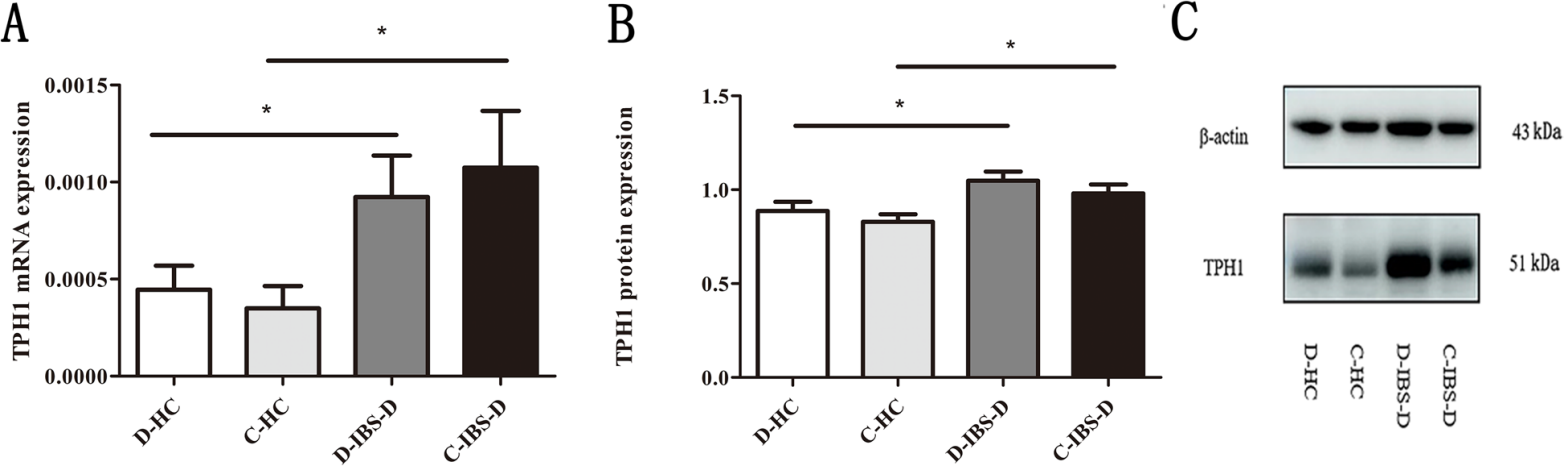
Expression of TPH1 in the IBS-D patients and HCs. **a** Quantitative real-time polymerase chain reaction analysis of Tph1 in the subjects. **b** Western blot analysis of Tph1 in the subjects. **c** Western blot analysis of Tph1 and β-actin in the subjects. Stripped blots were re-probed with β-actin. The data were normalized with housekeeping protein (β-actin). The full-length blots or gels are presented in Supplementary Fig. S4. (D-HC refers to the descending colon in HCs, D-IBS-D refers to the descending colon in IBS-D patients, C-HC refers to the caecum in HCs, C-IBS-D refers to the caecum in IBS-D patients). * *P* < 0.05

The numbers of EC cells in HCs were 5.10 ± 3.03 in the caecum and 7.32 ± 4.40 in the descending colon. In contrast, in IBS-D patients, the numbers of EC cells were 8.37 ± 5.78 in the caecum and 9.27 ± 5.96 in the descending colon. The number of EC cells increased significantly in the caecum of IBS-D patients compared to that in HCs (*P* = 0.029, Fig. 3). The transcriptional level of TPH1 was upregulated in the descending colon (*P* = 0.037, Fig. 4) and caecum (*P* = 0.026, Fig. 4) in IBS-D patients. In addition, western blot analysis revealed that the expression of TPH1 in IBS-D patients increased significantly in the descending colon (*P* = 0.02, Fig. 4, for the original image, see Additional file 4) and caecum (*P* = 0.013, Fig. 4, for the original image, see Additional file 4).

### Spearman correlation between the relative abundance of microbiota and clinical parameters

**Fig. 5:**
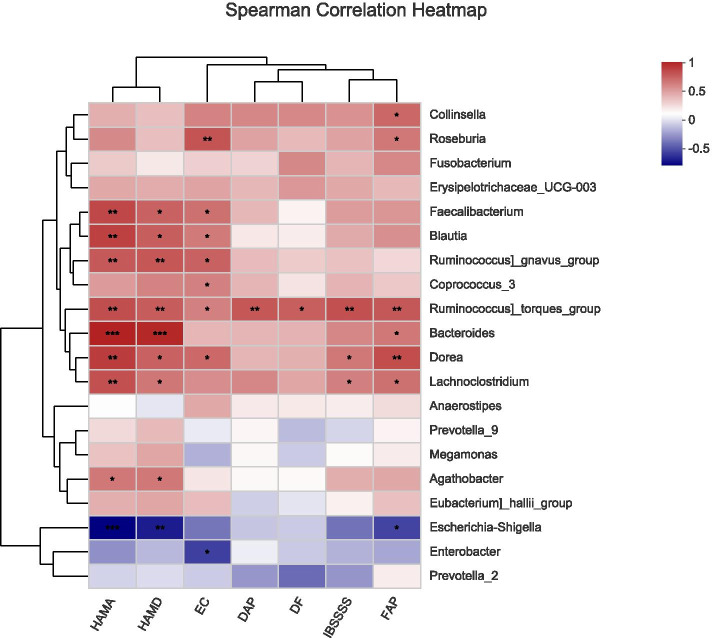
Relationships among the relative abundance of microbial communities at the genera level and clinical parameters in the descending colon of IBS-D patients based on the Spearman correlation analysis (HAM-A: Hamilton Anxiety Score; HAM-D: Hamilton Depression Score; EC: Enterochromaffin cell number; IBSSSS: IBS Symptom Severity System; DAP: Degree of abdominal pain; DF: Defecation frequency; FAP: Frequency of abdominal pain); ***P < 0.001; **P < 0.01; *P < 0.05

We further evaluated the correlation between the relative abundance of microbiota at the genus level and the number of EC cells, IBS Severity Scores (IBS-SSS), degree of abdominal pain, frequency of abdominal pain, frequency of defecation, HAM-A, and HAM-D in the descending colon of IBS-D patients. *Ruminococcus_torques_group*, which increased in IBS-D patients, was positively associated with HAM-A (*r* = 0.66, *P* = 0.004), HAM-D (*r* = 0.61, *P* = 0.009), EC cell number (*r* = 0.49, *P* = 0.047), IBS-SSS (*r* = 0.65, *P* = 0.004), degree of abdominal pain (*r* = 0.63, *P* = 0.007), frequency of abdominal pain (*r* = 0.63, *P* = 0.007), and frequency of defecation (*r* = 0.60, *P* = 0.011). *Dorea*, which increased in IBS-D patients, was positively associated with EC cell number (*r* = 0.57, *P* = 0.018), IBS-SSS (*r* = 0.52, *P* = 0.034), HAM-A (*r* = 0.72, *P* = 0.001), HAM-D (*r* = 0.59, *P* = 0.012), frequency of abdominal pain (*r* = 0.67, *P* = 0.003) (Fig. 5).

The results of Spearman correlation between the relative abundance of microbiota and the expression of TPH1 in the descending colon of IBS-D patients was shown in Additional file 5, and the results of Spearman correlation between the relative abundance of microbiota and clinical parameters in the descending colon of HCs were shown in Additional file 6. The OTU table and clinical parameters were shown in Additional file 7.

## Discussion

IBS-D has a significant influence on health-related quality of life, but its aetiology remains poorly understood. In this study, we investigated EC cells and gut microbiota in IBS-D patients and found links among gut microbiota, EC cells and IBS-D symptoms. Not only did the gut microbiota change in IBS-D patients, but also EC cells and TPH1 increased in IBS-D patients. Furthermore, *Ruminococcus_torques_group* and *Dorea* were positively correlated with EC cell number and IBS symptom scores. In addition, in a comparison between IBS-D patients and HCs, this study found microbiome variation in the caecum and descending colon.

Correlation analysis revealed a positive relationship between *Ruminococcus_torques_group* and the number of EC cells, so we speculated that the EC cell could be affected by gut microbiota. Mandić et al. showed that the increased expression of leucine-rich repeat-containing G-protein-coupled receptor 5, an EC stem cell marker, in mice monoassociated with *C. ramosum* than in GF mice. They suggested that gut microbiota can influence the development of colonic epithelial progenitor cells towards EC cells [21], which is consistent with our speculation. EC cells are triangular-shaped and the microvilli of the apex extend into the intestinal lumen to catch luminal stimuli. Metabolites produced by the microbiota, such as SCFAs, can act on host colonic EC cells and regulate the peripheral 5-HT mainly by affecting the expression of TPH1 [17]. Our study found that the expression of TPH1 and the relative abundance of *Ruminococcus_torques_group* increased in IBS-D patients, suggesting that the SCFA-producing *Ruminococcus_torques_group* might improve the expression of TPH1 and the synthesis of 5-HT.

Intestinal 5-HT plays an important role in intestinal motility and visceral sensation. Therefore, the increase in 5-HT levels in the gut may be related to the generation of IBS symptoms. Treatment with a TPH inhibitor or a 5-HT_3_ receptor antagonist can effectively relieve the symptoms of IBS [25]. As a chief source of 5-HT, the number of EC cells increased in the intestinal mucosa of IBS patients, particularly post-infectious IBS patients [26–28], which was consistent with our results. However, current treatments mainly target reducing the availability of 5-HT and do not influence the number of EC cells. It has been reported that physiological stress in early life promotes the differentiation of intestinal epithelial stem cells into enteroendocrine cells and induces EC cell proliferation in adults. In addition, infection and inflammation can also influence the number of EC cells [29], suggesting new therapeutic strategies for IBS.

The results of the mucosal microbiota assessment showed no differences in microbiota diversity between IBS-D patients and HCs. These findings were in line with those of some studies but were contradictory to some [16], which warrants further research. However, we found differences in relative abundance of genus level between IBS-D patients and HCs, suggesting that changes in gut microbiota may influence IBS-D patients. It may work through metabolites derived from microbiota including SCFAs, secondary bile acids, and tryptophan metabolites, which transmit the signals by interacting with enteroendocrine cells, such as EC cells. Some bacteria can ferment undigested carbohydrates to generate SCFAs, and gases, such as carbon dioxide, hydrogen, and methane, are also produced. These gases are reported to be partially responsible for abdominal pain and bloating in patients with IBS. In addition, protein residues can be fermented by bacteria to produce a variety of metabolites, including ammonia, organic acids, heterocyclic amides, phenols, and indoles, which affect intestinal epithelial metabolism and thus damage intestinal health. In addition to participating in the fermentation of various ingredients in food, the intestinal microbiota can also regulate the levels of neuroactive molecules, such as nitric oxide, substance P, and endocannabinoids, thereby affecting intestinal motility and visceral sensation. Gut microbiota are also related to mood disorders. Our results revealed that HAM-A and HAM-D were higher in patients with IBS-D than in HCs. In addition, we found that the relative abundance of some bacteria was associated with HAM-A and HAM-D. Inflammation resulting from exotoxins secreted by certain bacteria can be transmitted to the brain, leading to mental illness. Sudo et al. showed that the stress responsiveness of adults could be influenced by the absence of normal intestinal flora in early life, and these changes can be partly reversed by early colonisation of conventional gut microbiota [30]. The prophylactic effects of microbiota on anxiety and depression scores were also demonstrated, and faecal microbiota transplantation can affect anxiety-like and depression-like behaviours [31, 32]. Flora may be the future direction of therapy for the treatment of mood disorders.

In this study, we found that *Ruminococcus_torques_group* and *Dorea* increased in IBS-D patients, and correlation analysis showed a positive relationship between the relative abundance of *Ruminococcus_torques_group* and *Dorea* with IBS-SSS, indicating that *Ruminococcus_torques_group* and *Dorea* played important roles in the IBS-D patients. By resolving intestinal mucin, *Ruminococcus_torques_group* can damage the mucosal barrier and facilitate IBS. Lyra et al. revealed that *Ruminococcus_torques decr*eased in the group treated with probiotics compared with placebos [33], which suggested the pathogenicity of *Ruminococcus_torques* in IBS-D. Erja et al. assessed the faecal flora of IBS patients and found that the abundance of *Ruminococcus_torques_group* was positively correlated with the severity of bowel symptoms [34]. *Dorea* can promote the production of intestinal gases and contribute to abdominal discomfort. In addition, we found a decreased abundance of *Fusobacterium* and *Raoultella* in IBS-D patients. *Fusobacterium* has been reported to be related to colonic adenoma [35] and inflammatory bowel disease [36], and *Raoultella* may be associated with urinary tract infection and biliary tract disease [37]. However, its connection with IBS requires further investigation.

Intestinal flora in the caecum plays an important role in maintaining homeostasis in animal experiments. As the main fermentation site, the caecum serves as a “bag” structure between the colon and small intestine in mice. Portela-gomes et al. found that EC cells were mainly located in the caecum and gradually decreased from the proximal colon to the distal colon in rats. In this study, comparing the mucosa of the caecum and descending colon, there was no significant difference in the number of EC cells, TPH1 expression, and mucosal microbiota. Given that the human caecum is structurally quite different from that of rodents, its function may also be significantly different.

## Conclusions

This study showed a correlation between the number of EC cells, mucosal-associated microbiota, and IBS-D symptoms. *Ruminococcus_torques_group*, *Dorea*, and EC cells may be considered as targets for the treatment of IBS. Moreover, we should pay more attention to bacterial variations between the caecum and descending colon in IBS-D patients in future studies.

## Methods

### Participants and questionnaires

IBS-D patients and healthy controls (HCs) were recruited from the Gastroenterology Outpatient Department at Qilu Hospital, Shandong University. IBS-D patients were diagnosed by a gastroenterologist according to ROME IV criteria (recurrent abdominal pain on average at least 1 day per week in the last 3 months, associated with ≥2 of the following: related to defecation, associated with a change in stool frequency, associated with a change in stool form. The criteria must be met for the last 3 months, with symptom onset at least 6 months prior to diagnosis). The HCs were free of gastrointestinal symptoms and underwent routine health check-ups. The exclusion criteria included a history of any organic syndromes (celiac disease, tumours, inflammatory bowel disease, gastrointestinal infections, and psychiatric disorders), history of major abdominal surgery or use of probiotics, non-steroidal anti-inflammatory drugs, anti-inflammatory drugs, proton-pump inhibitors, prokinetics, anti-anxiety drugs, anti-depressants in the past 4 weeks, accompanying pregnancy, lactation, bladder irritation, chronic pelvic pain syndrome, dysmenorrhea, endometriosis, other painful gynaecological diseases, clotting disorder, and apparent mental disorders. Individuals with long-term alcohol consumption were excluded.

Information on subjects was collected by completing the questionnaires. The common information collected on HCs and IBS-D patients included sex, age, BMI, HAM-A [38], and HAM-D [39]. In addition, patients with IBS-D provided information on clinical symptoms and IBS-SSS [40]. The subjects underwent colonoscopy and three biopsies from the caecum and three biopsies from the descending colon were obtained using endoscopic biopsy forceps (MIN-BF-23; MICRO-TECH, Nanjing, China) during colonoscopy. One biopsy sample from the caecum and one from the descending colon were fixed in formalin. The other samples were stored immediately in liquid nitrogen.

### Immunofluorescence

The mucosa was fixed in formalin for 72 h and then embedded in paraffin. The paraffin block was cut into 4 μm sections using a cryostat and mounted onto glass slides. The sections were incubated in rabbit anti-CgA antibody (1:100; Abcam, Cambridge, UK) in a humidified box at 4 °C overnight and then with Alexa Fluor 488-conjugated anti-rabbit IgG (1:400; Abcam) in a black humidified box at room temperature for 1 h and fluoroshield mounting medium with DAPI (Abcam) for 5 min. Finally, the sections were observed under a fluorescence microscope (BX53, Olympus, Tokyo, Japan). EC cells were identified in the crypt epithelium and showed intense nuclear and cytoplasmic staining. The number of EC cells was quantified using methods described in previous studies [26, 27, 41]. A total of five non-overlapping high-power fields (final magnification 200×) were chosen, and two persons who were blinded to clinical data analyzed the number of EC cells.

### Quantitative real-time polymerase chain reaction

Total RNA was extracted using the AllPrep DNA/RNA/Protein Mini Kit (QIAGEN, Hilden, Germany) according to the manufacturer’s protocol. Quantitative real-time polymerase chain reaction (RT-PCR) was performed using the SYBR® Green Realtime PCR Master Mix (QPK-201, TOYOBO) in a 20 μL reaction volume containing the following reagents: 1 μL of cDNA preparation; SYBR® Green Realtime PCR Master Mix (TOYOBO); 7.4 μL PCR grade water, and 0.8 μM of forward and reverse primers. RT-PCR reactions were performed on an Applied Biosystems Stepone Real-Time PCR System (Thermo, Waltham, MA, USA) in triplicate using the following conditions: pre-denaturation, denaturation, annealing and extension (40 cycles): 95 °C for 1 min, 95 °C for 15 s, and 58 °C for 15 s, followed by 72 °C for 45 s. The specific primer sequences were as follows (5′ - 3′): TPH1: forward CTGGTTATGCTCTTGGTGTCTTTC, reverse TGCAAAGGAGAAGATGAGAGAATTTAC; β-actin: forward ATGATGATATCGCCGCGCTC, reverse CCACCATCACGCCCTGG.

### Western blotting

A total of 20 μg of extracted protein was separated using 10% SDS-PAGE and transferred to polyvinylidene difluoride membranes (Bio-Rad, Hercules, CA, USA). The membranes were then incubated in 5% skim milk for 2 h at room temperature. After that, the membranes were exposed to primary antibodies overnight at 4 °C and incubated for 1 h at room temperature using a secondary antibody. Finally, an enhanced chemiluminescence kit (Merck Millipore, Darmstadt, Germany) was used to detect the reaction products. The antibodies used in this study were as follows: rabbit anti-TPH1 (1:500; Abcam), anti-β-actin antibody (1:1000; Zhongshan Gold Bridge, Beijing, China), horseradish peroxidase-conjugated goat anti-rabbit IgG (1:1000; Zhongshan Gold Bridge), and goat anti-mouse IgG (1:1000; Zhongshan Gold Bridge). The results were quantified using Image J software (National Institutes of Health, Bethesda, MD, USA) to measure the grey values of the target and reference bands (β-actin). Then, the target/reference ratio was regarded as the relative expression level of the target.

### Mucosal microbiota assessment

An E.Z.N.A.® soil DNA Kit (Omega Bio-tek, Norcross, GA, USA) was used to extract microbial DNA from the biopsy samples of patients with IBS-D and HCs. The V3–V4 hypervariable regions of the bacterial 16S rRNA gene were amplified with primers 338F (5′- ACTCCTACGGGAGGCAGCAG-3′) and 806R (5′-GGACTACHVGGGTWTCTAAT-3′) using a thermocycler PCR system (GeneAmp 9700, ABI, USA). The PCR cycling parameters were as follows: 3 min of denaturation at 95 °C, 27 cycles of 30 s at 95 °C, 30 s for annealing at 55 °C, and 45 s for elongation at 72 °C, and a final extension at 72 °C for 10 min. The PCR products were separated on a 2% agarose gel and further purified using the AxyPrep DNA Gel Extraction Kit (Axygen Biosciences, Union City, CA, USA) and quantified using QuantiFluor™-ST (Promega, USA) according to the manufacturer’s protocol. All amplicons were sequenced on an Illumina MiSeq platform (San Diego, CA), as previously described [42]. The raw data files were demultiplexed, quality-filtered using Trimmomatic, and merged using FLASH. UPARSE (version 7.1 http://drive5.com/uparse/) was used to cluster the OTUs with a 97% similarity cutoff; UCHIME was used to identify and remove the chimeric sequences. The microbial composition and biodiversity were analysed. In addition, Venn diagrams were constructed to express the differences in OTUs and genera between the groups. Moreover, correlation analysis was conducted to show the relationship between the variables (EC cell number, HAM-A, HAM-D, IBS-SSS, degree of abdominal pain, frequency of abdominal pain, frequency of defecation) and microbial abundance.

### Statistical analysis

All the data were analysed using SPSS 22.0 and R 3.1.1. The data are expressed as mean ± SD for continuous variables and percentages for categorical variables. Differences between the two groups were evaluated using the independent-samples t-test or non-parametric test according to the data in a normal distribution. Categorical variables were analysed using the chi-square test. The correlation between variables and relative bacterial abundance was assessed using the Spearman correlation analysis. Statistical significance was set at *P* < 0.05.

## Supplementary Information

**Additional file 1.** Alpha and beta diversity in IBS-D patients and HCs. a. Microbial richness was assessed using the Chao index. No difference was observed between groups. b. Beta-diversity metrics were assessed using principal coordinates analysis and Bray-Curtis distance. No difference was observed between groups. (D-HC refers to the descending colon in HCs, D-IBS-D refers to the descending colon in IBS-D patients, C-HC refers to the caecum in HCs, C-IBS-D refers to the caecum in IBS-D patients).

**Additional file 2.** Relative abundances of the dominant phylum in the descending colon and caecum in HCs and IBS-D patients. No differences were observed between groups.

**Additional file 3.** Relative abundances of the dominant genera between the descending colon and caecum in HCs and IBS-D patients. a. Relative abundances of genera in IBS-D patients between the descending colon and caecum. No differences were observed between groups. b. Relative abundances of genera in HCs between the descending colon and caecum. No differences were observed between groups. (D-HC refers to the descending colon in HCs, D-IBS-D refers to the descending colon in IBS-D patients, C-HC refers to the caecum in HCs, C-IBS-D refers to the caecum in IBS-D patients).

**Additional file 4.** Original blot images of Fig. 4. (D-HC refers to the descending colon in HCs, D-IBS-D refers to the descending colon in IBS-D patients, C-HC refers to the caecum in HCs, C-IBS-D refers to the caecum in IBS-D patients).

**Additional file 5.** Relationships among the relative abundance of microbial communities at the genera level and the expression of TPH1 in the descending colon of IBS-D patients based on Spearman correlation analysis. (WBTPH1 refers to the expression of TPH1 at the protein level, PCRTPH1 refers to the expression of TPH1 at the gene level).

**Additional file 6.** Relationships among the relative abundance of microbial communities at the genera level and the clinical parameters in the descending colon of HCs based on Spearman correlation analysis. (WBTPH1 refers to the expression of TPH1 at the protein level, PCRTPH1 refers to the expression of TPH1 at the gene level; EC: Enterochromaffin cell number; HAM-A: Hamilton Anxiety Score; HAM-D: Hamilton Depression Score).

**Additional file 7.** The OTU table and clinical parameters.

## Data Availability

The dataset of 16S rRNA gene sequencing supporting the conclusions of this article is available in the National Center Biotechnology Information repository with accession code PRJNA762684.

## Abbreviations

IBS: Irritable bowel syndrome
IBS-D: Diarrhoea-predominant IBS
5-HT: 5-hydroxytryptamine
GF: Germ-free
EC: Enterochromaffin
CgA: Chromogranin A
TPH1: Tryptophan hydroxylase 1
SCFAs: Short chain fatty acids
HCs: Health controls
HAM-A: Hamilton anxiety scale
HAM-D: Hamilton depression scale
IBS-SSS: IBS Severity Scores
BMI: Body mass index
RT-PCR: Real time polymerase chain reaction
OTUs: Operational taxonomic units
