# Supplementary figures and images for: Involvement of mucosal flora and enterochromaffin cells of the caecum and descending colon in diarrhoea-predominant irritable bowel syndrome
Source: BMC Microbiol. 2021 Nov 13;21:316. doi: 10.1186/s12866-021-02380-2 (PMC8590216; doi:10.1186/s12866-021-02380-2)

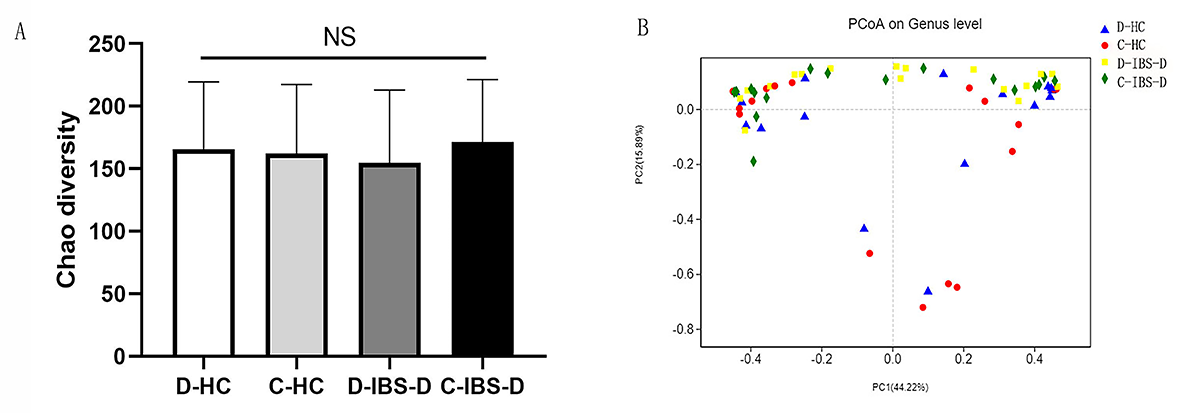

Supplement: Supplementary file 1 — Additional file 1. Alpha and beta diversity in IBS-D patients and HCs. a. Microbial richness was assessed using the Chao index. No difference was observed between groups. b. Beta-diversity metrics were assessed using principal coordinates analysis and Bray-Curtis distance. No difference was observed between groups. (D-HC refers to the descending colon in HCs, D-IBS-D refers to the descending colon in IBS-D patients, C-HC refers to the caecum in HCs, C-IBS-D refers to the caecum in IBS-D patients). [file 12866_2021_2380_MOESM1_ESM.tif]

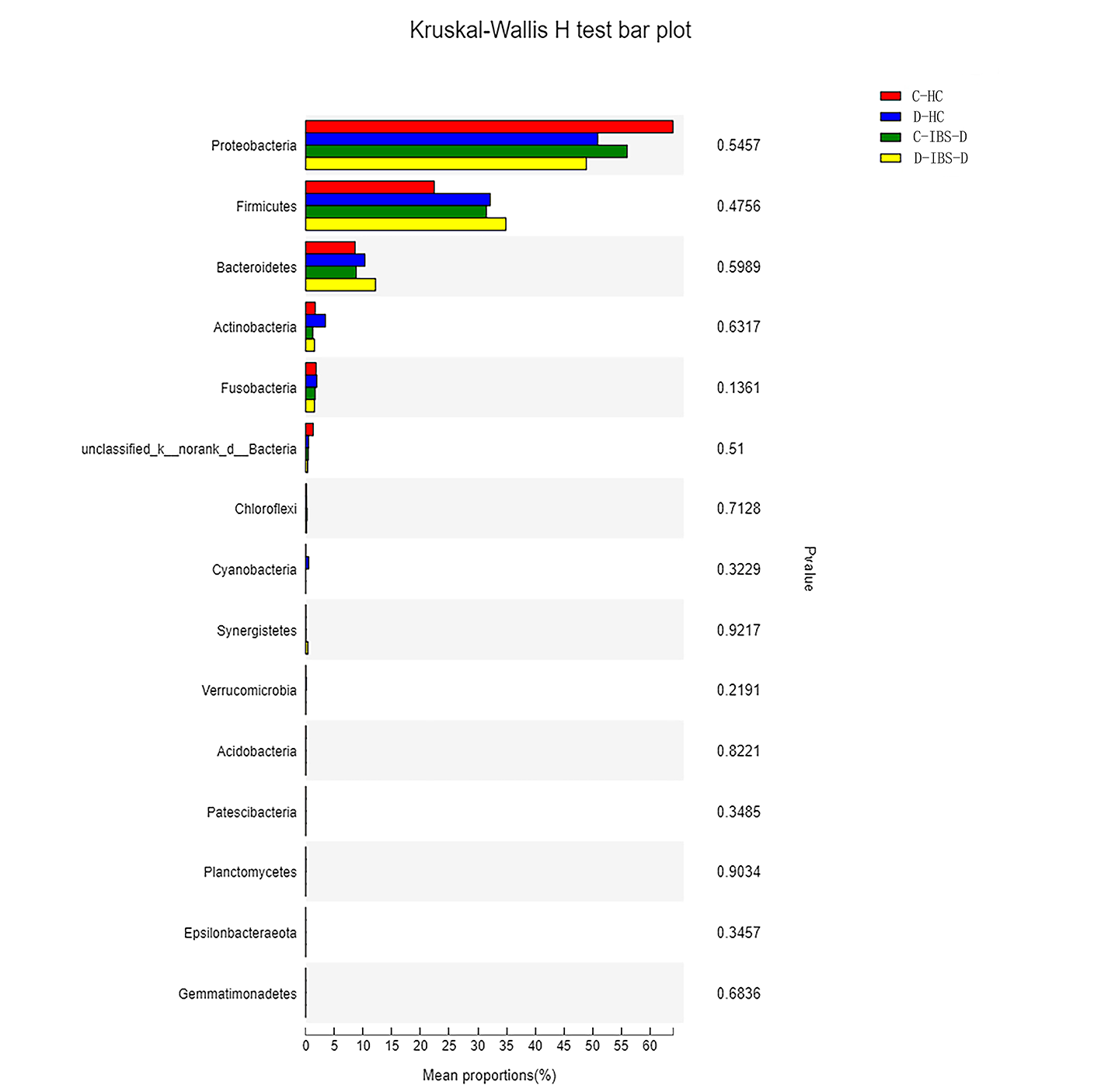

Supplement: Supplementary file 2 — Additional file 2. Relative abundances of the dominant phylum in the descending colon and caecum in HCs and IBS-D patients. No differences were observed between groups. [file 12866_2021_2380_MOESM2_ESM.tif]

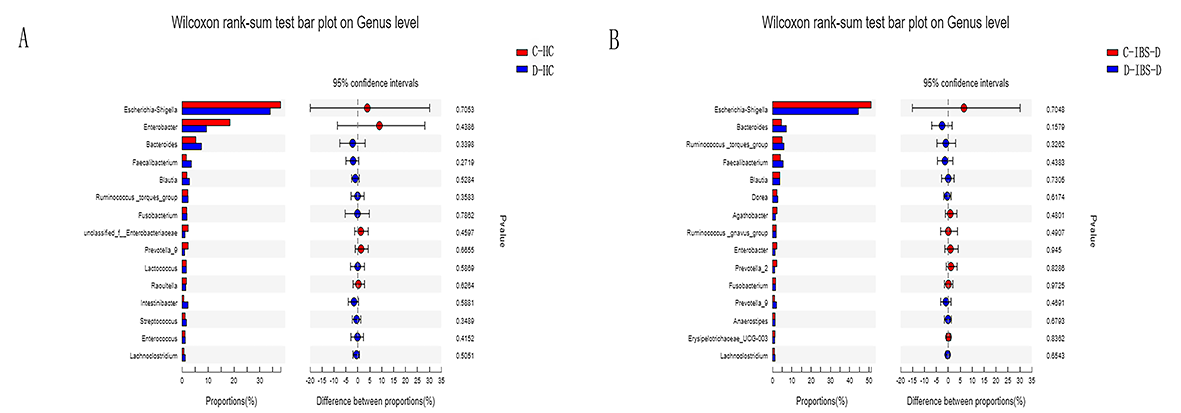

Supplement: Supplementary file 3 — Additional file 3. Relative abundances of the dominant genera between the descending colon and caecum in HCs and IBS-D patients. a. Relative abundances of genera in IBS-D patients between the descending colon and caecum. No differences were observed between groups. b. Relative abundances of genera in HCs between the descending colon and caecum. No differences were observed between groups. (D-HC refers to the descending colon in HCs, D-IBS-D refers to the descending colon in IBS-D patients, C-HC refers to the caecum in HCs, C-IBS-D refers to the caecum in IBS-D patients). [file 12866_2021_2380_MOESM3_ESM.tif]

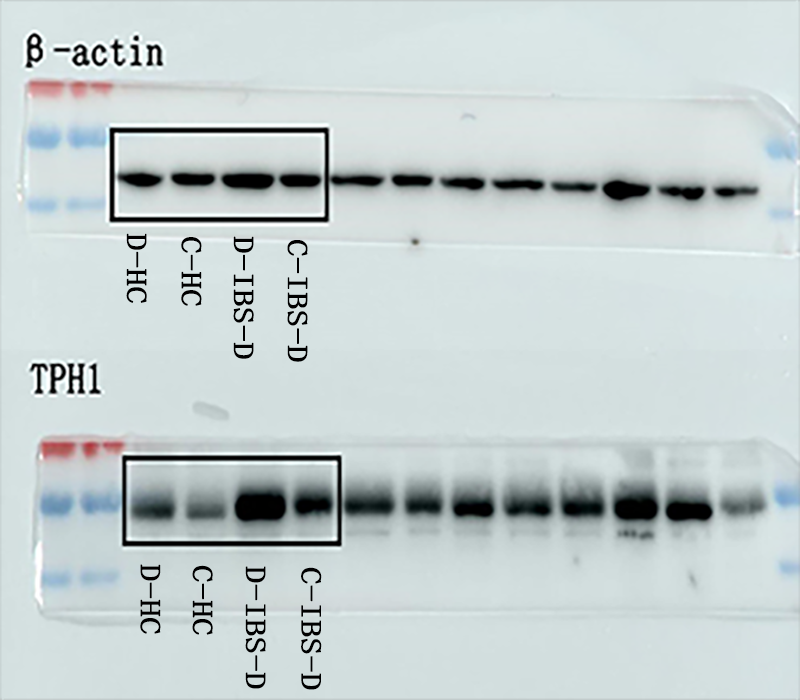

Supplement: Supplementary file 4 — Additional file 4. Original blot images of Fig. 4. (D-HC refers to the descending colon in HCs, D-IBS-D refers to the descending colon in IBS-D patients, C-HC refers to the caecum in HCs, C-IBS-D refers to the caecum in IBS-D patients). [file 12866_2021_2380_MOESM4_ESM.tif]

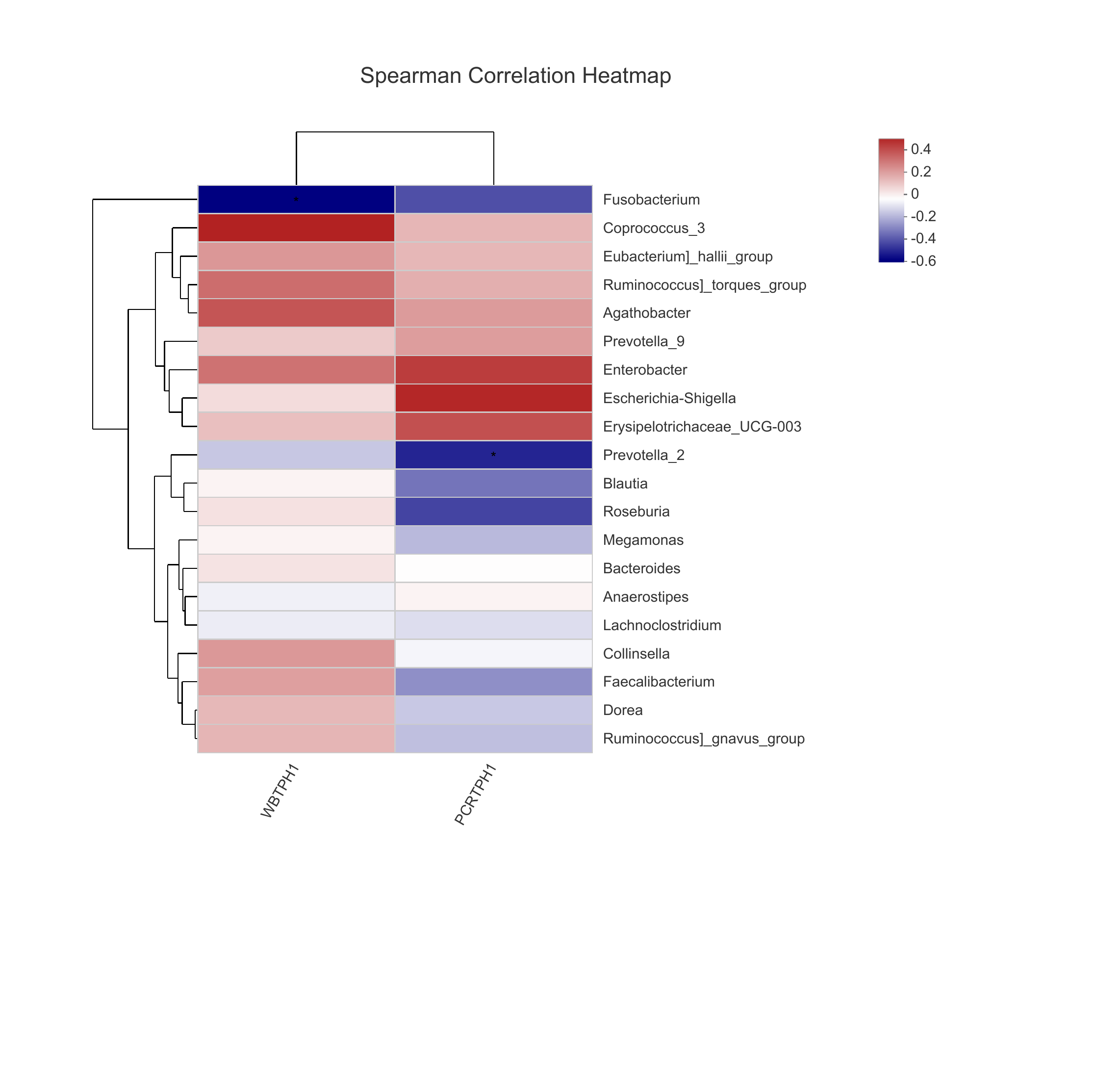

Supplement: Supplementary file 5 — Additional file 5. Relationships among the relative abundance of microbial communities at the genera level and the expression of TPH1 in the descending colon of IBS-D patients based on Spearman correlation analysis. (WBTPH1 refers to the expression of TPH1 at the protein level, PCRTPH1 refers to the expression of TPH1 at the gene level). [file 12866_2021_2380_MOESM5_ESM.tif]

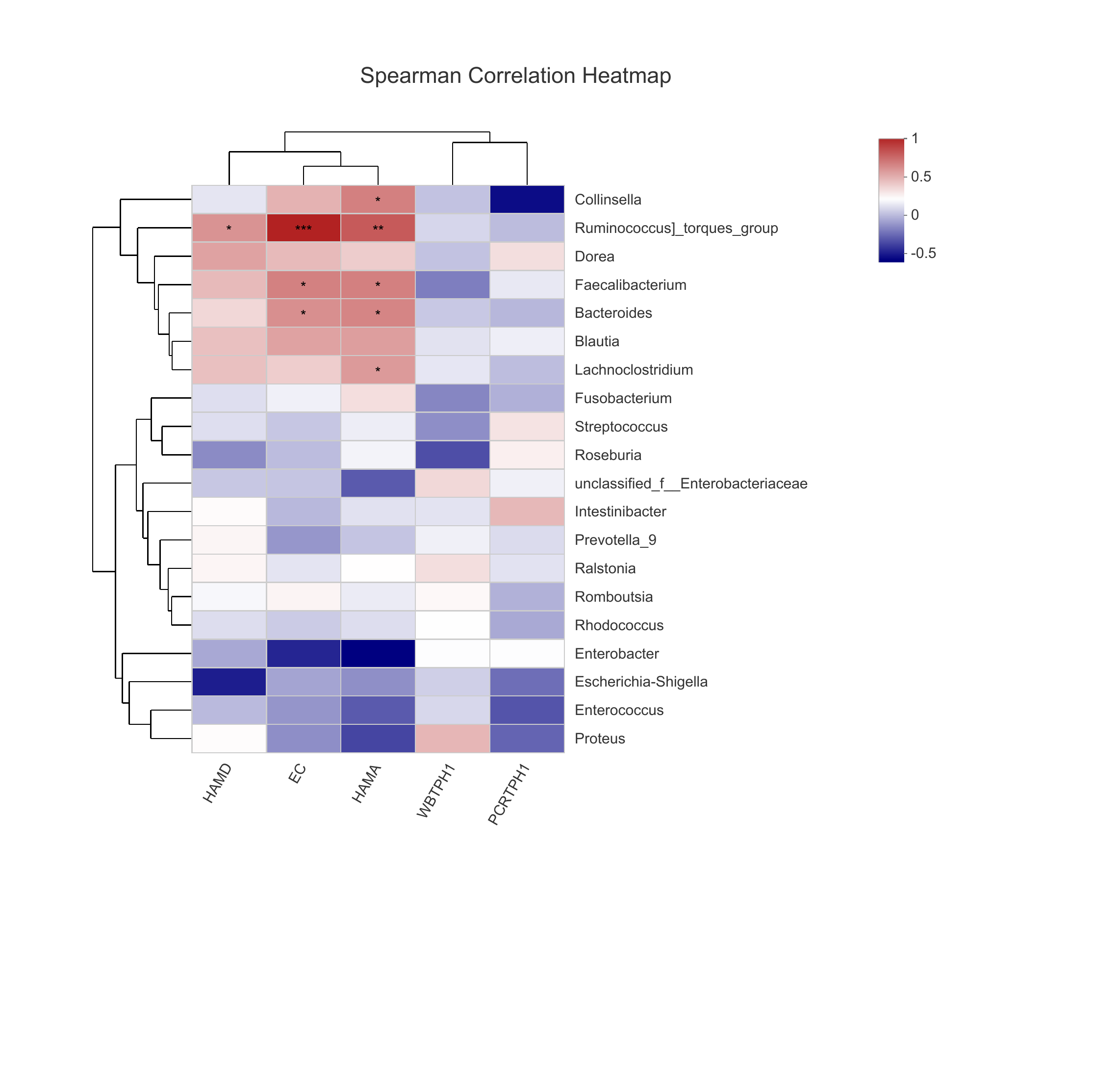

Supplement: Supplementary file 6 — Additional file 6. Relationships among the relative abundance of microbial communities at the genera level and the clinical parameters in the descending colon of HCs based on Spearman correlation analysis. (WBTPH1 refers to the expression of TPH1 at the protein level, PCRTPH1 refers to the expression of TPH1 at the gene level; EC: Enterochromaffin cell number; HAM-A: Hamilton Anxiety Score; HAM-D: Hamilton Depression Score). [file 12866_2021_2380_MOESM6_ESM.tif]
